# Topical 4‐Aminopyridine 5% in Male Androgenetic Alopecia: A Split‐Scalp Exploratory Series

**DOI:** 10.1111/jocd.70659

**Published:** 2026-01-04

**Authors:** Nicolò Rivetti, Claudio Zeccara

**Affiliations:** ^1^ Dermatology Outpatient Clinic Istituto Clinico Beato Matteo, Gruppo San Donato Vigevano Italy

Androgenetic alopecia (AGA) is the most common form of hair loss in men, affecting up to 80% of individuals during their lifetime [1]. Current FDA‐approved therapies, namely topical minoxidil and oral finasteride, can slow disease progression but do not induce de novo folliculogenesis [1]. Novel regenerative approaches are therefore of growing interest.

4‐Aminopyridine (4‐AP) is a voltage‐gated potassium channel blocker approved for multiple sclerosis. In murine wound models, 4‐AP has consistently promoted tissue regeneration: systemic administration accelerated closure and induced de novo folliculogenesis in excisional wounds [2], topical delivery improved burn healing with enhanced re‐epithelialization and follicle formation [3], and systemic treatment further facilitated burn repair by modulating inflammation, apoptosis, angiogenesis, and extracellular matrix remodeling [4]. Based on this rationale, we conducted a split‐scalp exploratory series to assess the potential of topical 4‐AP 5% in men with AGA.

We included 10 male patients with AGA, mean age 35.8 years (range 22–49), Hamilton–Norwood stage I–V, who were recruited and followed from January 2025 to May 2025. All had been on stable topical and/or oral minoxidil for ≥ 12 months. Patients on finasteride or dutasteride were excluded. Each patient underwent eight sessions of microneedling (dermaroller 1.5 mm, passes until pinpoint bleeding) at 2‐week intervals for 4 months. On the left scalp, 2 mL of a galenic 4‐AP 5% solution was applied; on the right scalp, 2 mL of saline served as intra‐patient control. Post‐treatment care included betadine disinfection and avoidance of minoxidil for 24 h. Clinical efficacy was assessed at baseline and after 4 months using global photography and standardized trichoscopy at four fixed scalp sites (temporal angle, baihui acupoint, hair whorl, and occipital tuberosity), as described by Wang et al. [5], Trichoscan analysis (hair density, shaft diameter, terminal/vellus ratio), and pull test.

All 10 patients completed the study with topical 4‐AP 5% solution, and no adverse events were reported. Global photography showed no visible improvement in density or hair caliber. Trichoscopy/Trichoscan revealed a mean hair density increase of +1.2 hairs/cm^2^ on the 4‐AP side vs. +0.8 hairs/cm^2^ on the saline side (NS), with no significant changes in terminal‐to‐vellus hair ratio or mean shaft diameter. The pull test remained unchanged. Representative images from two patients are shown in Figure 1, while patient characteristics and outcomes are summarized in Table 1.

**FIGURE 1:**
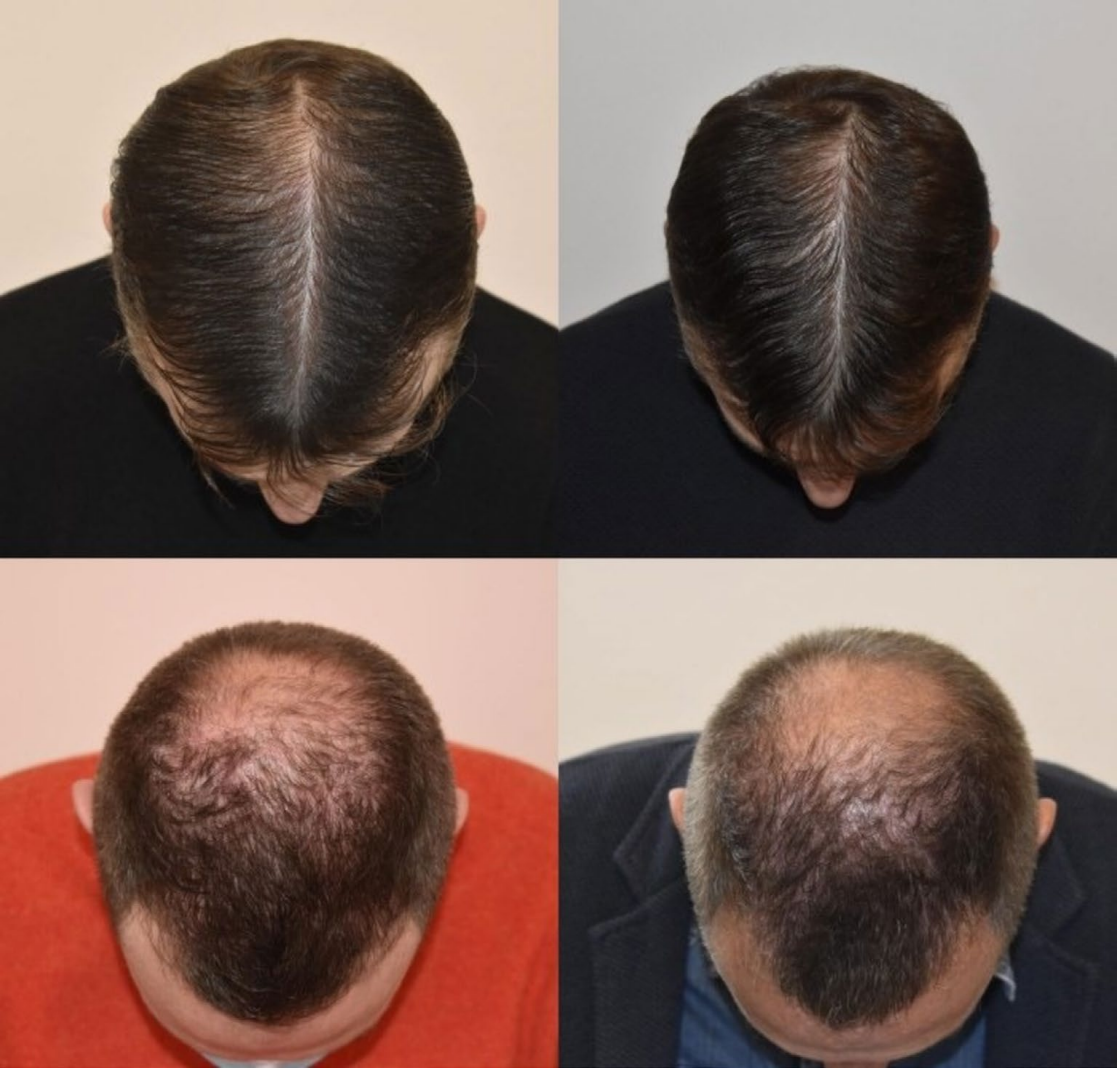
Representative global photographs of two male patients with androgenetic alopecia at baseline (left panels) and after 4 months of treatment (right panels). Each patient underwent a split‐scalp protocol with microneedling‐assisted topical 4‐aminopyridine 5% applied to one side of the scalp and saline to the contralateral side. No clinically meaningful differences in hair density or scalp coverage were observed between baseline and follow‐up or between treated and control sides.

**TABLE 1:** Baseline characteristics and trichometric outcomes of the 10 patients included in the split‐scalp exploratory series.

Patient ID	Age (years)	Hamilton–Norwood stage	Baseline hair density (hairs/cm^2^)	Change in hair density (4‐AP side)	Change in hair density (saline side)	Terminal/Vellus ratio change	Adverse events
1	28	II	172	+2	+1	0	None
2	33	III	160	+1	+1	0	None
3	41	IV	148	+1	0	0	None
4	25	II	180	+2	+1	0	None
5	39	V	142	0	+1	0	None
6	37	III	158	+1	+1	0	None
7	49	IV	145	+1	+1	0	None
8	22	II	176	+2	+1	0	None
9	35	III	155	+1	+1	0	None
10	42	IV	150	+1	+1	0	None

*Note:* Age, Hamilton–Norwood stage, baseline hair density, changes in hair density on the 4‐aminopyridine (4‐AP)–treated side and saline‐treated control side, terminal‐to‐vellus hair ratio variation, and adverse events are reported. Changes in hair density are reported as numerical variations from baseline as provided by trichoscopic analysis.

In our exploratory series, topical 4‐AP 5% did not show measurable benefit in male AGA. Notably, the beneficial effects reported in animal studies were observed in the setting of skin wounds. In mice, 4‐AP accelerated closure and induced de novo folliculogenesis in excisional wounds [2], improved healing and follicle formation in burn injuries when delivered topically [3], and enhanced burn repair when administered systemically through modulation of inflammation, apoptosis, angiogenesis, and extracellular matrix remodeling [4]. The difference between these regenerative wound‐healing contexts and the androgen‐driven follicular miniaturization of AGA may help to explain why the effects of 4‐AP did not translate into clinical benefit. Our findings therefore highlight a translational gap between wound‐healing–associated regeneration and androgenetic alopecia, underscoring the need for cautious extrapolation from preclinical models to human disease.

This exploratory series has several limitations. The split‐scalp design, while allowing intra‐patient comparison, may theoretically permit limited diffusion of the investigational product between treated areas. However, the absence of measurable differences between the 4‐aminopyridine and saline sides suggests that such diffusion did not materially influence the results. In addition, efficacy was assessed at the end of the 4‐month treatment period without extended post‐treatment follow‐up, thus limiting the evaluation of potential delayed or long‐term effects. Finally, the small sample size and exploratory nature of the study limit the generalizability of these findings.

In this 10‐patient, split‐scalp exploratory series, topical 4‐aminopyridine 5% solution combined with microneedling failed to improve clinical, dermoscopic, or trichometric parameters in androgenetic alopecia. These results do not support the use of 4‐AP in AGA, at least with the tested formulation and protocol.

## Author Contributions

Claudio Zeccara conceived the idea and contributed to study design. Nicolò Rivetti and Claudio Zeccara each recruited five patients and contributed to data acquisition. Nicolò Rivetti drafted the manuscript. Both authors contributed to data interpretation, revised the manuscript critically for important intellectual content, approved the final version, and agreed to be accountable for all aspects of the work.

## Ethics Statement

All patients provided written informed consent, and the study was conducted in accordance with the Declaration of Helsinki. Given the exploratory nature of this pilot experience and the absence of commercial sponsorship, institutional review board approval was not required according to local regulations.

## Consent

Written informed consent for the use of clinical photographs for publication purposes was obtained.

## Conflicts of Interest

The authors declare no conflicts of interest.

## Data Availability

The data that support the findings of this study are available on request from the corresponding author. The data are not publicly available due to privacy or ethical restrictions.
